# Efficacy and Safety of Simultaneous Integrated Boost Intensity-Modulation Radiation Therapy Combined with Systematic and Standardized Management for Esophageal Cancer

**DOI:** 10.3389/fsurg.2022.905678

**Published:** 2022-05-23

**Authors:** Wenzhao Deng, Xueyuan Zhang, Jingwei Su, Chunyang Song, Jinrui Xu, Xiaohan Zhao, Wenbin Shen

**Affiliations:** Department of Radiotherapy, The Fourth Hospital of Hebei Medical University, Shijiazhuang, China

**Keywords:** simultaneous integrated boost intensity-modulation radiation therapy, systematic and standardized management, dose-dependent intensity-modulated radiotherapy, esophageal cancer, efficacy and safe

## Abstract

**Objective:**

To analyze and compare the efficacy and safety of simultaneous integrated boost intensity-modulation radiation therapy (SIB-IMRT) combined with systematic and standardized management for esophageal cancer.

**Methods:**

From January 2012 to January 2019, 200 patients with esophageal cancer who received radical chemoradiotherapy in our hospital were treated with lymphatic drainage area radiation prevention combined with systematic and standardized management. According to difference in radiotherapy methods, the patients were divided into local lesion 92 patients treated with simultaneous integrated boost intensity-modulation radiation therapy (SIB-IMRT) combined with systematic standardized management (SIB-IMRT group), and late course boost intensity-modulation radiation therapy (LCB-IMRT) combined with systematic standardized management 108 patients (LCB-IMRT group). The short-term eficacy of the two groups were compared. The dose volume parameters of the organ in danger are evaluated based on the dose volume histogram. The related adverse reactions during chemoradiotherapy were compared between two groups. The local control rate and survival rate were compared between the two groups.

**Results:**

The recent total effective rates of rats in the SIB-IMRT group and LCB-IMRT group were 95.65% and 90.74%, respectively, and there was no significant difference between the two groups (*p *> 0.05). The mean doses to left and right lung, heart and spinal cord in the SIB-IMRT group were significantly lower than that in the LCB-IMRT group (*p *< 0.05). There was no significant difference in the incidence of adverse reactions such as radiation esophagitis, radiation pneumonitis, radiation tracheitis, gastrointestinal reaction and bone marrow suppression between the SIB-IMRT group and LCB-IMRT groups (*p *> 0.05). The one-year and three-year overall survival rates in the SIB-IMRT group and LCB-IMRT groups were 82.61%, 42.39% and 77.78%, 34.26%, respectively, and the median survival times were 38 and 29 months, respectively. The local control rates in the SIB-IMRT group and LCB-IMRT group in one and three years were 84.78%, 56.52% and 75.93%, 41.67%, respectively. The 3-year local control rate in the SIB-IMRT group was higher than that in the LCB-IMRT group (*p *< 0.05), but there was no significant difference in the 1-and 3-year overall survival rates between the two groups (*p *> 0.05).

**Conclusion:**

SIB-IMRT combined with systematic and standardized management in the treatment of esophageal cancer can reduce the amount of some organs at risk and improve the local control rate of the lesion.

## Introduction

In recent years, with the constant changes in people’s daily life and diet structure, the incidence of esophageal cancer is increasing year by year, and it is more likely to occur in the elderly (1). Early esophageal cancer is mainly treated by surgery, but most patients are in the advanced stage at the time of diagnosis, mainly by concurrent chemoradiotherapy. In addition, radiotherapy is one of the main methods to treat esophageal tumors for patients who can not receive surgical treatment (2–4). Esophageal carcinoma is a dose-dependent malignant tumor. The local control rate of esophageal cancer is positively ralated to the dose of radiotherapy, but many important organs are close to the periphery of the esophagus. Considering that the normal area around the tumor tissue has a certain dose limitation, it is difficult to obtain a more suitable dose distribution in the total tumor volume with conventional radiotherapy (5). Traditional radiotherapy techniques, which is simulated by esophageal barium meal radiography, may not be able to irradiate the tumor tissue and/or there is a low dose area in the tumor tissue, and it is difficult to increase the local dose of esophageal lesions due to the tolerance dose limitation of surrounding normal tissues and organs (6, 7).

With the extensive development of intensity-modulated radiotherapy, the five-year survival rate of esophageal cancer has been significantly improved compared with conventional two-dimensional radiotherapy. At present, the commonly used intensity-modulated radiation therapy commonly used in clinic includes conventional dose intensity-modulated radiation therapy, locally synchronous integrated intensity-modulation radiation therapy (SIB-IMRT), etc. The simultaneous integrated boost intensity-modulation radiation therapy allows different doses to be given to different irradiation areas in the same treatment, which not only increases the irradiation dose of tumor bed area, but also does not increase the tolerance dose of the surrounding normal tissues, and shorten the whole treatment time (8–10).

In the past, traditional clinical management only paid attention to the patients’s disease progress and treatment status, and explained the disease and treatment status to the patients orally, while ignoring the influence of psychological and spiritual factors on the disease, which made the treatment effect difficult to achieve clinical expectations. Systematic and standardized management is a newly emerging mode in recent years. It adheres to the service concept of “people-oriented” under the mode of psychological-physiological-social medicine, and implements a series of management interventions according to patients’ specific condition, psychology and mental state, so as to meet the their psychological, physiological and social needs and improve their quality of life (11). The purpose of this study was to explore the curative effect of SIB-IMRT combined with systematic and standardized management for the treatment of esophageal cancer, and to provide reference for the individualized plan for comprehensive radiotherapy for esophageal cancer.

## Data and Methods

### General Information

From January 2012 to January 2019, a total of 200 patients with esophageal cancer who received radical chemoradiotherapy in our hospital were selected, and treated with prophylactic irradiation in lymphatic drainage area combined with systematic and standardized management. According to different radiotherapy methods, the patients were divided into local lesion 92 patients treated with SIB-IMRT combined with systematic standardized management (SIB-IMRT group), and LCB-IMRT combined with systematic standardized management 108 patients (LCB-IMRT group). Inclusion criteria: The first treatment was confirmed as esophageal squamous cell carcinoma by pathology; Fluid food can be fed before radiotherapy, with KPS score ≥70; Receiving radical chemoradiotherapy or radical radiotherapy (dose ≥50 Gy); No signs of esophageal bleeding or perforation before radiotherapy; No other history of malignant tumor; All patients signed informed consent of radiotherapy or chemoradiotherapy. Exclusion criteria: Endoscopic report of a superficial tumor with no obvious esophageal lesion on CT images or endoscopic ultrasonography showing only invasion of the lamina propria and submucosa; Well-differentiated cancer; Esophagus has perforation signs; Esophageal surgery has been performed; There is distant organ metastasis; Incomplete follow-up information or follow-up failure.

### Research Methods

#### Radiotherapy

All the patients received radiotherapy through 6MV-X-ray of Elekta accelerator (Meda, Sweden). The patient took the supine position, and lay flat on the positioning bed. The positioning membrane was used to fix the body position. The scans were performed under a CT analog positioner. The scanning range from the cricoid cartilage to the celiac trunk was selected according to the different lesion sites. The whole neck should be scanned for patients with cervical esophageal cancer. The scanning layer was 5 mm thick and was transmitted digitally to the Treatment Planning System (TPS) for three-dimensional image reconstruction. Diagnostic criteria for a primary tumor were a thickness of the esophageal wall >0.5 cm or a diameter of the airless esophageal lumen >1.0 cm. The diagnostic criteria of metastatic lymph nodes were as follows: short diameter of lymph nodes in mediastinum ≥1.0 cm, and long diameter of paraesophageal, tracheoesophageal groove, pericardial horn and abdominal lymph nodes ≥0.5 cm.

Gross tumor volume delineation: The gross tumor volume (GTV) consists of the primary esophageal tumor and the metastatic lymph nodes (GTVnd); is delineated separately if the metastatic lymph nodes are far from the esophageal lesion); The clinical target volume (CTV) is the axial abduction of the primary tumor GTV by 0.8–1.0 cm, and up and down by 2.0–3.0 cm, with appropriate modifications based on the anatomical barrier. CTVnd refers to the uniform outward expansion of GTVnd of 0.5–0.8 cm in all directions. The planned target volume (PTV) and PTVnd were expanded evenly by 0.5–1.0 cm over the CTV and CTVnd.

The prescription doses of SIB-IMRT (different doses in different target areas but with the same irradiation times) were 58.05–65.10 Gy for 95%PTV and 95%PTVnd, with 28–31 times, and a single dose of 1.95–2.15 Gy; The prescription dose of LCB-IMRT was 46–54 Gy for 95%PTV in 23–27 times with a single dose of 1.8–2.0 Gy, and 10–16 Gy for PTV and PTVnd in 5–8 times to 58–66 Gy in 29–33 times with a single dose of 2 Gy.

#### Systemic Normative Management and Treatment

Pre-radiotherapy preparation: Health education and individual assessment before radiotherapy can improve patients’ cognition of the disease and radiotherapy, realize the importance of radiotherapy and self-care, correct patients’ biased understanding of radiotherapy, meet patients’ psychological needs, alleviate patients’ adverse psychological reactions, and ensure the smooth progress of chemotherapy treatment. The contents of education included: concept of radiotherapy, preparation before radiotherapy, adverse reactions of radiotherapy, cooperation during radiotherapy, psychological guidance, etc. Individual evaluation contents include patients’ condition, psychological condition, family and social support, disease cognition, disease behavior ability, etc. In addition, we should also pay attention to the psychological counseling of patients’ families to get their understanding and cooperation.

Quality control in radiotherapy: The problems existing in patients during interviews were taken as the guidance for patients to formulate systematic and standardized management plan. Patients were regularly screened for risk during chemotherapy, and nutritional support treatment was given based on the screening results in combination with patients’ relevant blood indicators. After chemotherapy, the responsible nurse completes the continuous evaluation list of radiotherapy education, including the systematic evaluation and treatment of patients’ adverse reactions, such as gastrointestinal reactions and bone marrow suppression, and puts forward the corresponding nursing measures and continuous improvement.

Quality management after chemotherapy: A personal file was established when the patient was discharged from hospital. In addition to the basic information such as the patient’s condition, home address, and contact information, the interview records were also recorded in the file. Subsequently, for each follow-up visit, the time and method of follow-up visit, patients’ various problems and improvements need to be continuously recorded in personal files. All patients were followed up for 3 consecutive years, one month after the end of treatment, every 3 months for 2 years, and every 6 months for 3 years. A patient communication area was established through WeChat, and the WeChat contact information was established for each patient. Face-to-face conversation guidance was provided to patients during outpatient re-examination or when they came to our hospital for radiation therapy. Contact the relevant physicians for the individual problems of the patients, and formulate the improvement plan for the patients. According to the patients’ cultural level and understanding ability, health education manuals, videos, pictures and case presentations were used to guide the patients to cultivate healthy behavior and self-care ability.

### Observation Indicators

#### Comparison of Short-Term Efficacy of Patients Between the Two Groups

The short-term efficacy is evaluated one month after radiotherapy, and the lesion retraction is evaluated according to the reexamination of chest CT changes before and after treatment. The short-term efficacy is divided into complete response, partial response, stability, and progression. Complete remission: the known lesions completely disappeared, and no new lesions appeared, which lasted for at least 4 weeks; Partial remission: the sum of the largest diameters of lesions decreased by ≥30% and maintained for at least 4 weeks; Stability: the sum of the maximum diameters of target lesions is reduced to the standard of partial remission, or increased to the standard of disease progression; Disease progression: The sum of the maximum diameters of target lesions increased by at least ≥20%, and their absolute values increased by at least 5 mm, or new lesions appeared. Total effective rate = (complete response + partial response)/total cases × 100%.

According to the dose-volume histogram, the dose-volume parameters of organs in danger were evaluated, including the average radiation doses of left and right lungs, heart and spinal cord.

Comparison of adverse reactions related to radiltherapy and chemotherapy between the two groups. The patients’ condition were recorded weekly during radiotherapy, and evaluated by RTOG standard classification according to the existence of acute radiation injury in patients who were followed up after radiotherapy.

#### Comparison of Local Control Rate and Survival Rate Between the Two Groups

Survival time was calculated from the date of diagnosis until death or end of follow-up. The deadline for follow-up is January 2022. Within 6 months after radiotherapy, local uncontrolled lesions appeared at the original lesion site, and tumor recurrence is defined as lesion lasting more than 6 months.

### Statistical Methods

SPSS22.0 software was used for processing. The measurement data of the experimental data were expressed as mean standard **± **deviation $\left(\overset{¯}{x}\pm s\right)$, and t test was used for pairwise comparison. The enumeration data were expressed as (%) and the comparison was conducted by *χ*^2^ test. Kaplan-Meier method was used for survival analysis. The Kaplan-Meier method was used to calculate the local control rate and survival rate at 1 and 3 years. The test level was *α* = 0.05, and *p *< 0.05 indicated that the difference was statistically significant.

## Results

### Patients with General Data Comparison

There was no significant difference in general information such as gender, age and lesion length between the two groups (*p *> 0.05). See Table 1.

**Table 1:** Comparison of general data of patients.

Project	SIB-IMRT group (*n* = 92)	LCB-IMRT group(*n* = 108)	*t/χ* ^2^	*p*
Gender			0.466	0.495
Male	63	69		
Woman	29	39		
Age (years)	60.28 ± 6.37	59.92 ± 6.18	0.405	0.686
Lesion length (cm)	4.71 ± 1.16	4.92 ± 1.59	1.051	0.295
Food intake			0.060	0.806
Common food	27	30		
Semi/liquid food	65	78		
Hoarseness			1.047	0.306
Yes	5	10		
No	87	98		
Lesion site			0.322	0.570
Cervical segment	8	12		
Thoracic segment	84	96		
Clinical t staging			0.029	0.864
T1	33	40		
T2–4	59	68		
Clinical n-staging				
N0	32	38	0.004	0.953
N1–2	60	70		
Chemotherapy			0.363	0.547
Yes	55	60		
No	37	48		

### Comparison of Short-Term Efficacy Between the Two Groups

The recent total effective rates of the SIB-IMRT group and the LCB-IMRT group in the same period were 95.65% and 90.74%, respectively, and there was no significant difference between the two groups (*p *> 0.05). See Table 2.

**Table 2:** Comparison of short-term efficacy between the two groups (*n*,%).

	Complete remission	Partial response	Stable condition	Disease progression	Total effective rate
SIB-IMRT group (*n* = 92)	31	57	3	1	95.65%
LCB-IMRT group (*n* = 108)	28	70	6	4	90.74%
*χ* ^2^					1.841
*p*					0.175

### Comparison of Exposure of Endangered Organs Between the Two Groups

**Figure 1:**
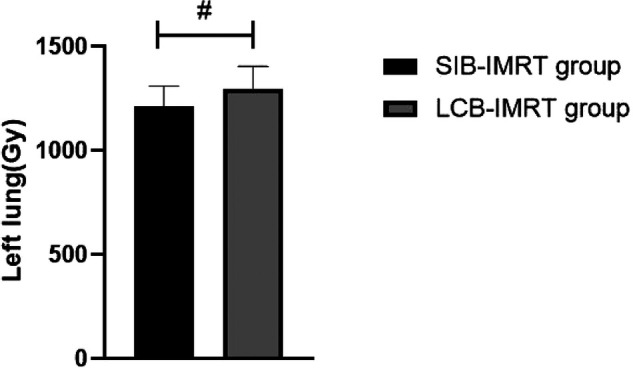
Comparison of average left lung exposure between the two groups. Note: Compared with the LCB-IMRT group, ^#^*p *< 0.05.

**Figure 2:**
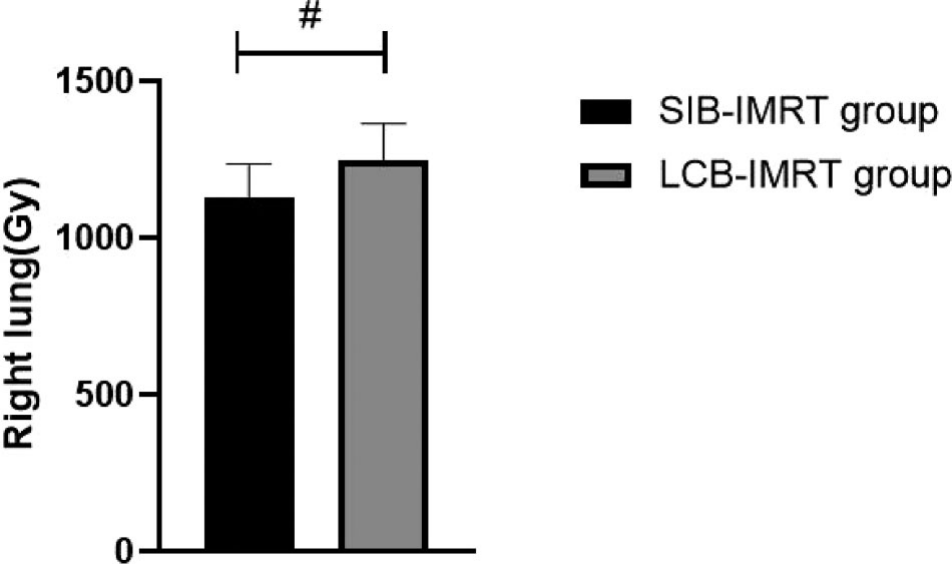
Comparison of average right lung exposure between the two groups. Note: Compared with the LCB-IMRT group, ^#^*p *< 0.05.

**Figure 3:**
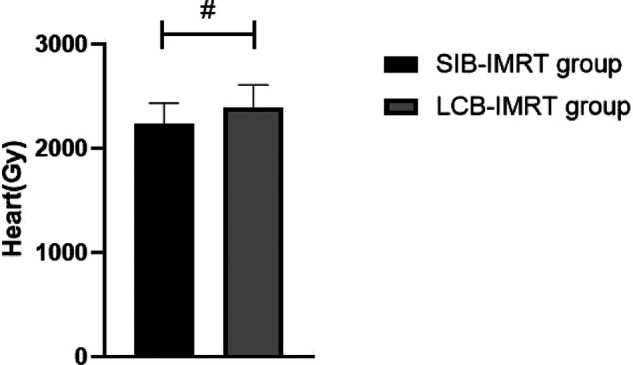
Comparison of average heart exposures between the two groups. Note: Compared with the LCB-IMRT group, ^#^*p *< 0.05.

**Figure 4:**
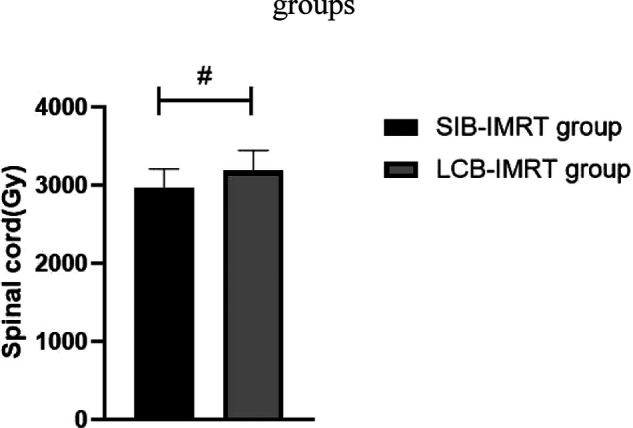
Comparison of average spinal cord exposures between the two groups. Note: Compared with the LCB-IMRT group, ^#^*p *< 0.05.

The average radiation doses to left and right lung, heart and spinal cord in the same period of the SIB-IMRT group were significantly lower than those in the LCB-IMRT group, and the differences were statistically significant (*p *< 0.05). See Figures 1–4.

### Comparison of the Incidence of Adverse Reactions Between the Two Groups

**Figure 5:**
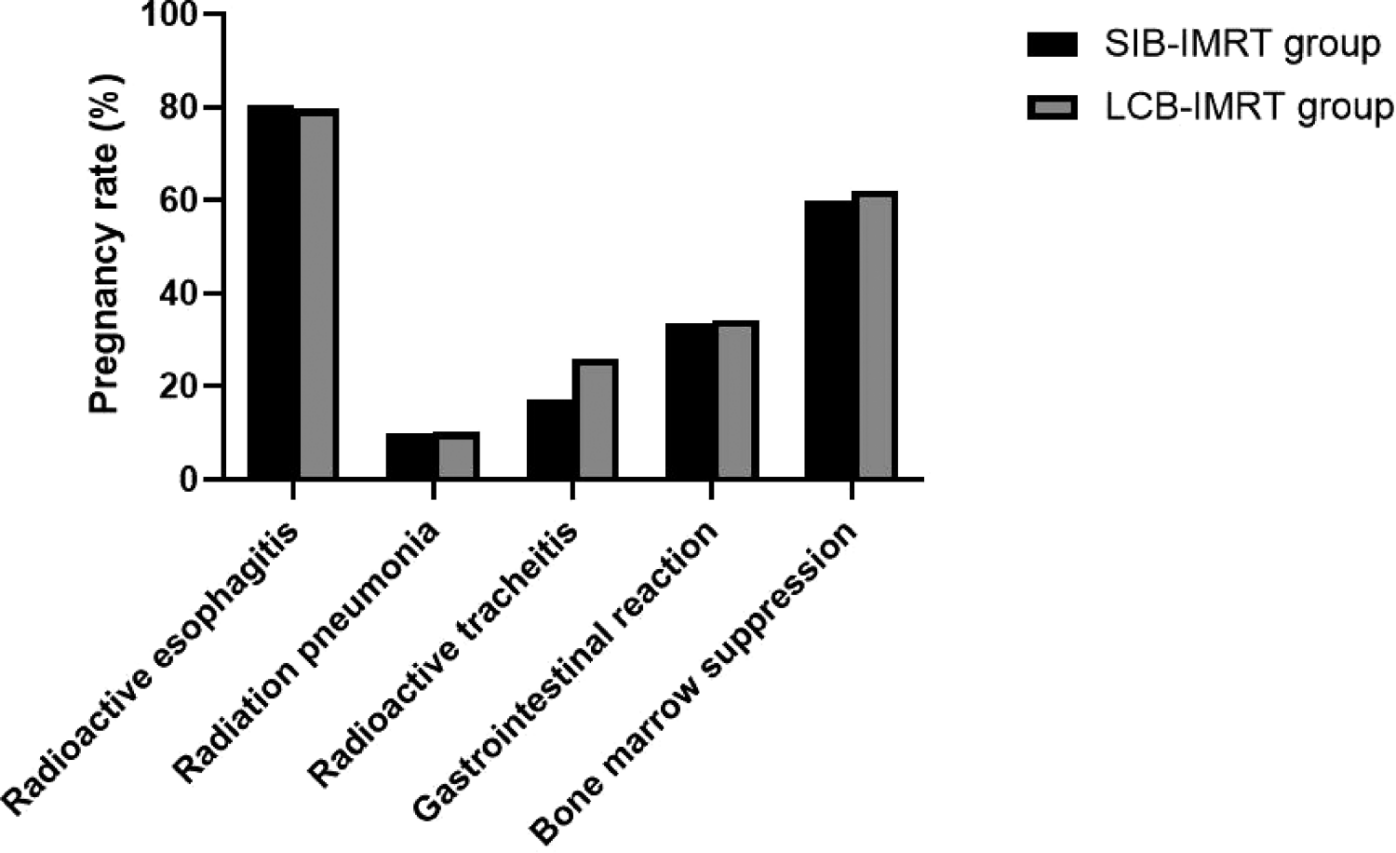
Comparison of the incidence of adverse reactions between the two groups.

The incidences of radiation esophagitis, radiation pneumonitis, radiation tracheitis, gastrointestinal reaction and bone marrow suppression in the SIB-IMRT group and the LCB-IMRT group were 80.43% and 79.63%, 9.78% and 10.19%, 17.39% and 25.93%, 33.70% and 34.26%, 59.78% and 62.04%, respectively. There was no significant difference in the incidence of adverse reactions between the two groups (*p *> 0.05). See Figure 5.

### Comparison of Long-Term Efficacy Between the Two Groups

**Figure 6:**
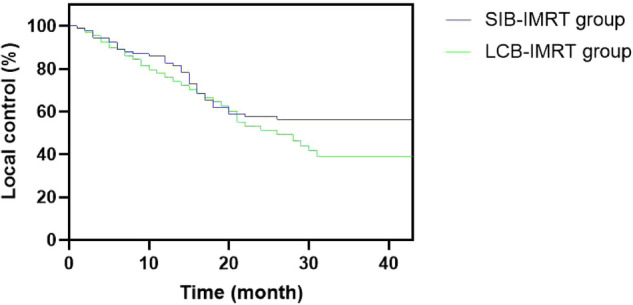
Comparison of local control curves between the two groups.

**Figure 7:**
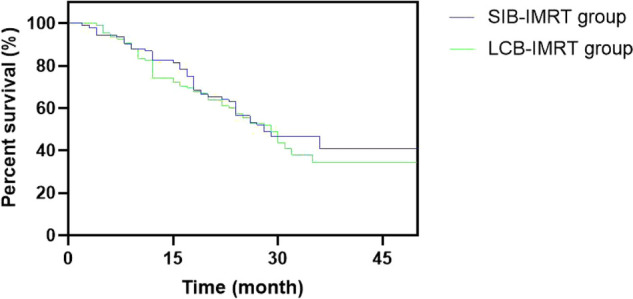
Comparison of survival curves of patients between the two groups.

The one-year and three-year overall survival rates in the SIB-IMRT group and the LCB-IMRT group were 82.61%, 42.39% and 77.78%, 34.26%, respectively, and the median survival times were 38 and 29 months, respectively. The local control rates in the SIB-IMRT group and the LCB-IMRT group in one-year and three-year were 84.78%, 56.52% and 75.93%, 41.67%, respectively. The three-year local control rate in the SIB-IMRT group was higher than that in the LCB-IMRT group (*p *< 0.05), but there was no significant difference in the one-year and three-year overall survival rates between the two groups (*p *> 0.05). See Figures 6 and 7.

## Discussion

Esophageal cancer is one of the common digestive tract cancers in China. Most patients have no obvious clinical symptoms in the early stage. Therefore, when seeing a doctor, most patients are in the advanced stage, and most of them are elderly patients. And surgical radiotherapy and chemotherapy are the main treatment methods (12, 13). Although intensity-modulated radiation therapy has been increasingly used in the treatment of esophageal cancer in recent years, local uncontrolled and recurrence are still the main methods of treatment failure. Therefore, it may be an important method to improve the curative effect of esophageal cancer by using the maximum dose in the tumor area and reducing the irradiated dose to the surrounding normal tissues (14–16).

The target area of the cervical and upper thoracic esophagus is large and irregular in shape, which makes it difficult to complete a single plan of conformal radiotherapy technology, requiring segmental irradiation. In addition, due to the limitation of normal tissue dose, the local dose in the target area is relatively low (17). However, the local dose increase of esophageal cancer should be carefully considered, especially the single dose which is prone to perforation and bleeding. In addition, the esophagus is located in the chest cavity, adjacent to the lung, heart, spinal cord and other important organs, with limited local stress increase (18–20). In this study, we compared the short-term efficacy of patients in the two groups who received SIB-IMRT and LCB-IMRT. The results showed that there was no significant difference between the two groups. However, the average exposure of left and right lung, heart and spinal cord in patients treated with SIB-IMRT was lower than that of LCB-IMRT. The SIB-IMRT not only has the advantages of highly conformal dose distribution of intensity-modulated radiotherapy and effective protection of surrounding normal organs, but also has the advantages of high efficiency, accuracy, high biological effect and satisfactory dose distribution in the target area by using field-in-field irradiation technology, so as to reduce the dose to some dangerous organs, especially to better protect heart and lung (21–24).

This study showed that there was no significant difference between the two groups in the incidence of adverse reactions such as radiation esophagitis, radiation pneumonia, radiation tracheitis, gastrointestinal reactions and bone marrow suppression. Concurrent chemotherapy will inevitably aggravate treatment-related adverse reactions, especially acute radiation esophagitis, which often leads to the delay or interruption of radiotherapy plans (25). Compared with the traditional LCB-IMRT, the SIB-IMRT can achieve the purpose of receiving different doses in different target areas at the same time, shorten the timeof radiotherapy and improve the intensity and efficiency of treatment. From the perspective of radiobiology, the increase of a single dose can make the total tumor volume obtain a higher equivalent biological dose, thereby improving the radiobiological effects and reducing the radiation dose to the surrounding organs (26–29).

Chemotherapy is an effective method for the clinical treatment of advanced esophageal cancer. However, as a strong stressor, it will directly invade the patient’s immune system, causing serious trauma to the body, resulting in negative psychology such as anxiety and depression, further affecting the patient’s immune system, reducing the body’s immunity to tumor cells, and even failing to successfully complete chemotherapy. Systematic and standardized management of patients with esophageal cancer undergoing chemotherapy can meet the nursing requirements at all stage of chemotherapy. At the same time, paying attention to individualzed nursing, strengthens psychological nursing, improving patients’ compliance with treatment, and having strong clinical applicability are of great significance to ensure the normal progress of patients’ chemotherapy (30).

In this study, systematic and standardized management was selected, while the advantages of SIB-IMRT were fully utilized, in an attempt to kill the subclinical lesions and well control the local lesion and metastatic lymph nodes within the limited treatment time. The results showed that the local control rate during the same period of three years by SIB-IMRT group was superior to that by the LCB-IMRT group. These results suggest that SIB-IMRT may be a better option for improving long-term local control. Although the overall survival rates of the patients in the two groups are similar, the survival curve of the concurrent dosed intensity-modulated radiotherapy shows a significant increase trend compared with the sequential dosed intensity-modulated radiotherapy, and the survival curve remains at about 50%. The effect of SIB-IMRT in radical chemoradiotherapy for esophageal cancer was confirmed.

To sum up, the SIB-IMRT group can reduce the exposure of some dangerous organs and has certain advantages in improving the local control rate of esophageal cancer. However, this study still has certain limitations. The individualized treatment plan with sub-layers needs long-term results of multi-centers and more cases in the future to be canfirmed.

## Data Availability

The original contributions presented in the study are included in the article/supplementary material, further inquiries can be directed to the corresponding author/s.
